# Processing Ultrasonic Data by Coda Wave Interferometry to Monitor Load Tests of Concrete Beams

**DOI:** 10.3390/s18061971

**Published:** 2018-06-19

**Authors:** Ernst Niederleithinger, Xin Wang, Martin Herbrand, Matthias Müller

**Affiliations:** 1 Bundesanstalt für Materialforschung und -prüfung (BAM), Unter den Eichen 87, 12205 Berlin, Germany; xin.wang @bam.de; 2 RWTH Aachen University (now WTM Engineers GmbH), Templergraben 55, 52062 Aachen, Germany; martin.herbrand@outlook.de; 3 Bundesanstalt für Straßenwesen (BASt), Bruederstr. 53, 51427 Bergisch Gladbach, Germany; muellerm@bast.de

**Keywords:** ultrasound, concrete, monitoring, coda wave interferometry

## Abstract

Ultrasonic transmission measurements have been used for decades to monitor concrete elements, mostly on a laboratory scale. Recently, coda wave interferometry (CWI), a technique adapted from seismology, was introduced to civil engineering experiments. It can be used to reveal subtle changes in concrete laboratory samples and even large structural elements without having a transducer directly at the place where the change is taking place. Here, several load tests until failure on large posttensioned concrete beams have been monitored using networks of embedded transducers. To detect subtle effects at the beginning of the experiments and cope with severe changes due to cracking close to failure, the coda wave interferometry procedures had to be modified to an adapted step-wise approach. Using this methodology, we were able to monitor stress distribution and localize large cracks by a relatively simple technique. Implementation of this approach on selected real structures might help to make decisions in infrastructure asset management.

## 1. Introduction

Monitoring of concrete structures is of utmost importance to cope with the ageing of infrastructure in many countries [1]. A large variety of sensors and data processing methods is used to assess loads, structural health, load capacity, and remaining lifetime. As all available technologies have their limitations and most of them react to more than one influence factor, a monitoring system should be carefully designed, adapted to the specific structure and the loads affecting it, and consist of more than one sensing technology.

Ultrasonic transmission measurements have been used for decades to monitor concrete elements, mostly on a laboratory scale. Their main application is to estimate concrete strength from wave velocities as described e.g., in European standards [2]. Ultrasonic transmission is also used in the assessment of freeze-thaw resistance of concrete samples [3]. Recently, they were successfully applied in concrete fatigue experiments [4]. Data evaluation is mostly performed as determination of time of flight and velocity. Unfortunately, the sensitivity of this technique is limited, as distances between transmitter and receiver are short (and so is transit time) and quite low frequencies (25–150 kHz) must be used due to the inherent scattering properties of concrete. 

Recently, coda wave interferometry (CWI), a technique adapted from seismology, was introduced to civil engineering experiments [5]. It is based on the comparison of the late part of waveforms (“coda”), containing multiple reflections and scattering events to reference data. As the travel paths and transit times are much longer and the waves may have traveled more than once through the region of interest, this technique can be used to reveal subtle changes in concrete laboratory samples and even large structural elements. Several variants of CWI exist and are discussed later in this paper. A review of the application of CWI on concrete is given in [5]. As other techniques, CWI has its limitations—loss of directionality, lack of discrimination between wave types or limited ability to pinpoint the location of changes (structural ones as cracks or local alteration of material parameters) when using just one transmitter/receiver pair. In addition, CWI algorithms break down if changes in the material exceed a certain, often-unforeseeable limit. A way to overcome this issue is described in this paper.

CWI is not limited to single transmitter receiver configurations. When using networks of transducers, localization of changes is possible up to a certain extent. While most approaches are complex and computationally intensive [6,7], simplified techniques have been proposed [8]. In this paper, we show a very simple alternative.

In our opinion, the two main advantages of using ultrasonic waves for monitoring are: first, the ability to detect changes at certain places in a structural element without having to place transducers exactly there. Distances up to five meters between transducers are possible, but closer placement leads to higher resolution. Second, ultrasonic waves are sensitive to many types of changes (which might be considered as a disadvantage at the same time), as stress, temperature, moisture and any kind of damage causes structural changes as cracks. In this study, we show an application to monitor stress distribution and cracking in concrete beams loaded until failure. Some preliminary results of this study have already been published in [9]. 

## 2. Coda Wave Interferometry

### 2.1. Principle

**Figure 1:**
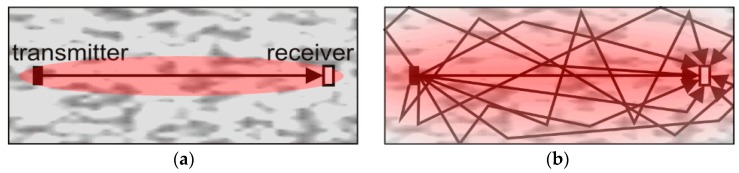
Principle of ultrasonic transmission measurements, propagation paths, and areas of influence (red). (**a**) Direct wave (time of flight); (**b**) multiple scattering (coda).

Coda Wave Interferometry has been shown to be very sensitive in several studies [10]. CWI was applied to monitor water saturation in sandstone [11], thermally induced velocity change [11,12], and stress changes in a real concrete bridge in a practical environment [13]. It was also used to detect and locate cracks in concrete [6]. As multiply scattered waves travel much longer than direct or single reflected ones (Figure 1), they are much more sensitive to weak perturbations of the medium.

The unperturbed wave field $u_{u}\left(t\right)$ can be written as a sum of the waves that propagate along the multiple scattering trajectories T in the medium, where *t* denotes time and $A_{T}\left(t\right)$ is the wave that has propagated along trajectory T:

(1) $$\;u_{u}\left(t\right)=\sum A_{T}\left(t\right)$$

when the medium changes over time, the dominant effect is a change $\tau_{T}\;$in the arrival times of the waves that propagate along each trajectory *T*, so that the perturbed wave field $u_{p}\left(t\right)$ is given by:

(2) $$\;u_{p}\left(t\right)=\sum A_{T}\left(t-\tau_{T}\right)$$

By comparing the coda obtained from a fixed source and receiver pair in two different states, it is possible to monitor weak velocity variations in the medium as well as some other parameter changes. The most common way to quantify these changes are correlation coefficient (CC) and velocity change. The cross correlation is the basis of the CWI. It can be made in both frequency and time domain [14]. It is used to compute the degree of similarity of two signals:

(3) $$CC\left(t,\;\delta t\right)=\frac{\int_{t-T}^{t+T}u_{u}\left(t^{\prime }\right)u_{p}\left(t^{\prime }+\delta t\right)dt^{\prime }}{\sqrt{\int_{t-T}^{t+T}u_{u}{}^{2}\left(t^{\prime }\right)dt^{\prime }\int_{t-T}^{t+T}u_{p}{}^{2}\left(t^{\prime }\right)dt^{\prime }}}$$

**Figure 2:**
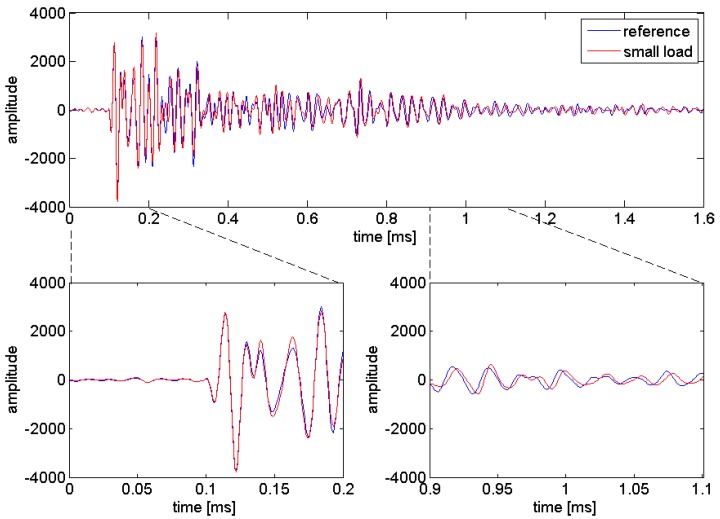
Ultrasonic signals (All ultrasonic signals in Section 2 are acceleration data in arbitrary units, as recorded by the data acquisition system. The real (absolute) values are not important for the issues discussed here) for the same configuration and sample, without load (reference) and subjected to a small load. While time of flight almost remains constant, the late part of the signal (coda) shows a significant phase change. Experimental data from the load test is described later.

An example of experimental signals presented in Figure 2 shows the sensitivity of coda wave following a velocity change. The blue curve represents the unperturbed wave field $u_{u}\left(t\right)$, which is chosen as the reference signal, and the red curve represents the perturbed wave field $u_{p}\left(t\right)$.

Other ways to describe changes in a medium by consecutive ultrasonic measurements are, for example, tracking changes in the amplitude or calculating parameters as magnitude-squared coherence [15].

### 2.2. Preprocessing

Depending on the hardware set-up (see Section 3.1), data acquisition parameters and the signal-to-noise ratio preprocessing of each ultrasonic time series might be required. In our case, we recorded 100 samples before signal transmission (“pretrigger”) to determine DC offsets and noise level. This part of the signal is deleted before CWI evaluation. In addition, the following steps were carried out:

- Offset compensation
- Crosstalk removal (replacing the first samples by zeros)
- Bandpass filtering (passband 10–150 kHz)

**Figure 3:**
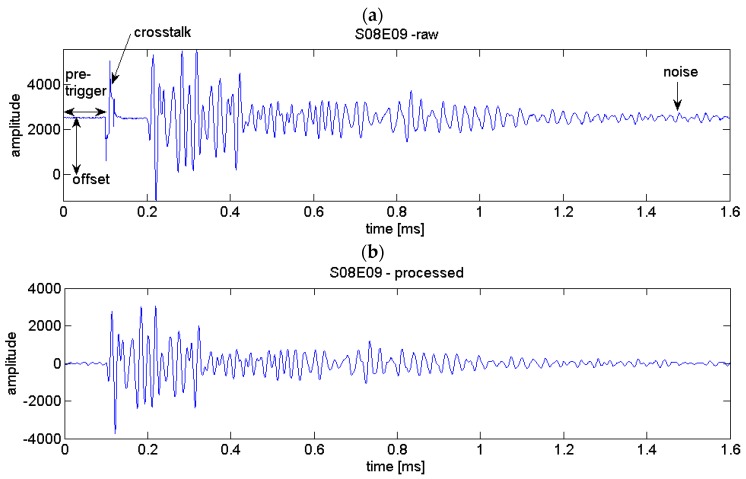
Example of an ultrasonic signal from the experiments described in Section 3 (**a**) before and (**b**) after data processing.

Figure 3 shows an example of an ultrasonic time series, before and after data processing.

### 2.3. CWI Algorithms to Determine Velocity Change

#### 2.3.1. Doublet Technique

The doublet technique was the first approach for velocity change determination and was widely used in geophysics [16]. The principle is to determine the velocity change by evaluating the shift of acoustic field $\delta t_{v}$ at time t:

(4) $$\frac{\delta v}{v}=\frac{\delta t_{v}}{t}$$

**Figure 4:**
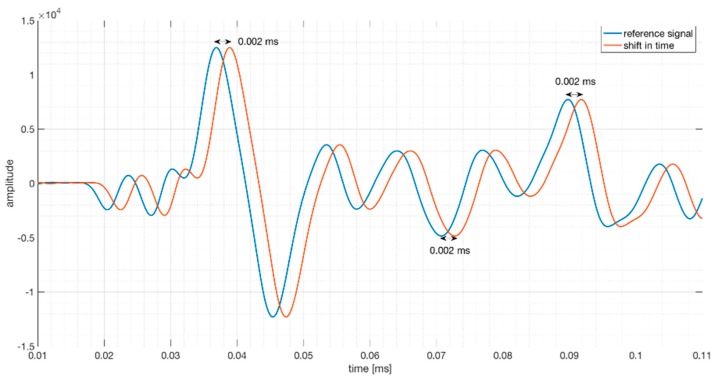
Two signals with a shift of 0.002 ms in time.

The doublet method assumes that the shift in time is (almost) constant in a certain time window. (Figure 4).

The cross correlation is now a function of the shift δt (Equation (3)). The value of $\delta t=t_{MAX}$, which maximizes the CC(δt), is the shift between the two signals corresponding to center time t of a chosen time window. $t_{MAX}$ is the average of all the $\delta t_{v}$ associated with each moment in this chosen time window [16].

#### 2.3.2. Stretching Method

**Figure 5:**
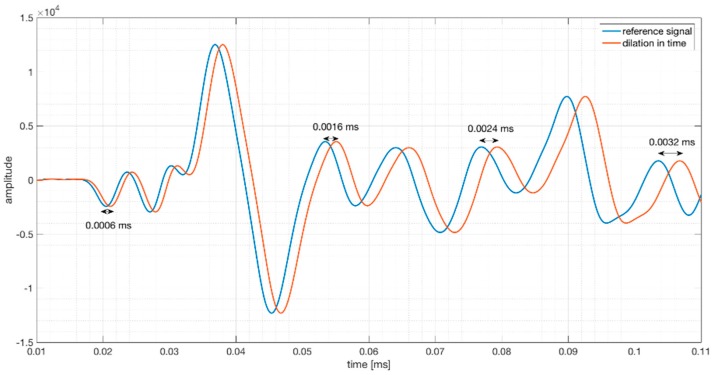
Signal with a dilatation factor (α = 0.08) in time; the time difference increases over time.

The stretching method [17] considers that the result of the velocity change is a dilation in time (Figure 5). The compression or dilation of the reference signal with a dilation rate α simulates an increase or decrease of propagation velocity. Firstly, a reference signal is chosen and is stretched with different dilation rates. Then, the cross correlation between the new signal and all the stretched signals are calculated. Velocity change dv/v is determined by choosing α, which maximizes the cross correlation.

(5) $$u_{P}\left(t\right)=u_{u}\left(t\left(1+\alpha \right)\right),$$

The cross-correlation coefficient (CWI-CC) at this point indicates whether the velocity change is linear and homogeneous (CWI-CC = 1) or local or nonlinear changes have taken place in the medium (CWI-CC < 1). The stretching method was used to evaluate the experiments in this study. 

#### 2.3.3. Taylor Series Expansion Method

The Taylor series expansion method [18] is another method to determine the stretch factor. Equation (5) is expanded as a first order Taylor series:

(6) $$\;u_{p}\left(t\right)=u_{u}\left(t\left(1+\alpha \right)\right)=u_{u}\left(\left(t+t\alpha \right)\right)=u_{u}\left(t\right)+u_{u}{}^{\prime }\left(t\right)\alpha +o\left(t\alpha \right)$$

when the high order o(tα) is ignored, α can be calculated by:

(7) $$\alpha \approx \frac{u_{p}\left(t\right)-u\left(t\right)}{u_{u}{}^{\prime }\left(t\right)t}$$

This method has higher computational efficiency because the stretch factor can be directly obtained from the signals without calculating cross-correlation coefficients. However, the precision and accuracy are limited because the high order terms in the calculation equations are ignored.

### 2.4. Step-Wise CWI Procedures

**Figure 6:**
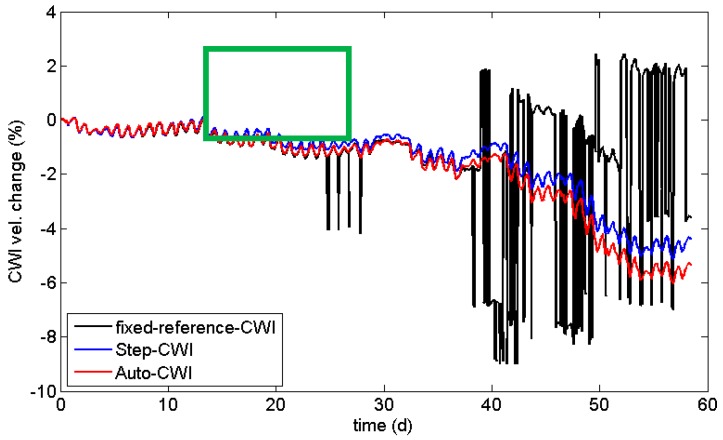
Velocity change calculated by the CWI stretching method using a fixed reference (CWI), moving reference (Step-CWI), and an auto-moving reference (Auto-CWI). Data from a temperature-affected reinforcement corrosion experiment on a concrete sample. Green box marks detail shown in Figure 7.

**Figure 7:**
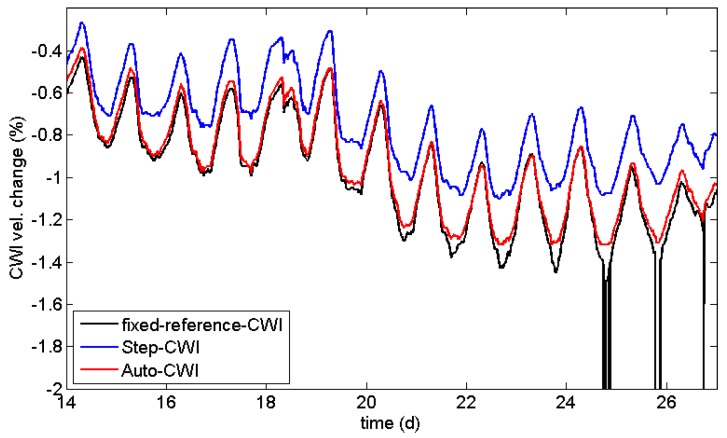
Detail of data shown in Figure 6. The general trend is similar for all procedures, but Step-CWI (blue line) shows a significant offset due to rounding errors and standard CWI (black line) is prone to fail at some point.

**Figure 8:**
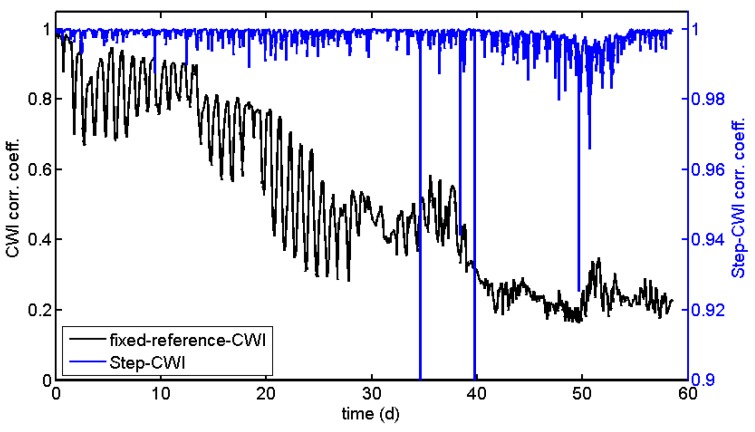
CWI cross-correlation coefficients for the same experiment as in Figure 6.

As a standard procedure, CWI properties are calculated using one or more fixed reference traces, usually recorded at the beginning of the actual experiment. This works well, if changes in the material do not exceed a certain, hardly predictable limit. If this limit is exceeded (i.e., the evaluated parts of the waveform are shifted by more than half a wavelength or the waveforms are completely changed by structural damage), the velocity changes are not meaningful anymore, while the correlation coefficients might still be useful. This is shown in Figure 6 and Figure 7 (black line) using data recorded in an experiment on a reinforced concrete sample subjected to accelerated corrosion (not related to the load test described later). The experiment suffered from high daily temperature changes in a laboratory. After about 25 days, the procedure starts to fail for a few data points. After 38 days, it breaks down, well before the actual failure (cracking) of the sample. At this point in time, the waveform is too different to the reference, resulting in low values of the CWI cross-correlation coefficient (Figure 8). 

One way to overcome this issue is to calculate stepwise changes by using just the previous measurement as reference (Step-CWI). Even experiments with large changes can be monitored by CWI. The stepwise velocity changes can be multiplied in an appropriate way to provide information on the cumulative change, relative to the beginning of experiment (blue line in Figure 6). These values should match the velocity changes of the standard CWI procedure, but might be affected by rounding errors (Figure 7). However, the cross-correlation coefficients cannot be cumulated, as this procedure is non-unique. Nevertheless, values deviating significantly from 1 are indications for sudden changes in the material (Figure 8). These sudden changes correspond to anomalies in the curve for the fixed reference procedure. However, long-term trends are not visible in CWI cross-correlation coefficients for Step-CWI.

In this study, the step-wise version of coda wave interferometry was used for data evaluation.

To minimize the effect of rounding errors on CWI velocity changes, one could change the reference in larger, fixed intervals. We propose the use of an automated, data-driven procedure by selecting the new reference if the CWI cross-correlation coefficient exceeds a certain threshold (Auto-CWI, red lines in Figure 6 and Figure 7). This results in less frequent switches to a new reference when the velocity change is small (reducing rounding errors, see Figure 7) and timely reference switches when changes are large. The difference in velocity change is quite significant at the end of the experiment shown in Figure 6. For quantitative analysis of velocity changes, the Auto-CWI is recommended, while the step-CWI might be sufficient for qualitative analysis. 

The CWI cross-correlation coefficients calculated by the Auto-CWI procedure are not quite useful for monitoring and thus not displayed in Figure 8. One could use cross-correlation of the original waveforms (stepwise or with a fixed reference) instead.

All procedures work independently of the CWI algorithm (Section 2.3) implemented. Thresholds and other parameters should be adapted to the CWI algorithms and the experimental set-up. 

## 3. Load Experiments on Concrete Beams

### 3.1. Load Test Set-Up

In the frame of a large-scale project organized and funded by the German road research institute (Bundesanstalt für Straßenwesen, BASt), several dual span posttensioned girders with varying cross-sections were subjected to load tests until failure. The purpose of these tests was to identify load capacity reserves, as current codes are based on the result of single span experiments. In addition, the detailed distribution of stress and strain as well as some details of the failure mechanism are not yet fully understood for certain geometries [19]. This project is carried out as new codes for load capacity calculations of existing bridges, introduced in Germany recently.

**Figure 9:**
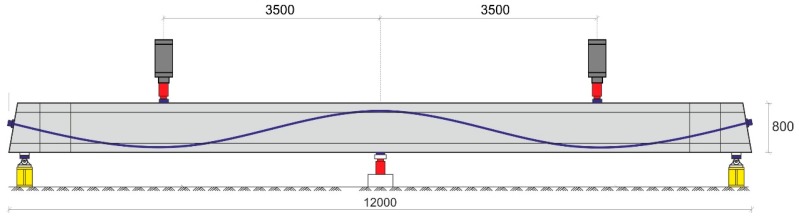
Sketch of load test setup for girder DLT 1.3 (dimensions in mm). Blue line: tendon duct.

The girders are 12 m in length and 0.8 m in height. The cross-section was either rectangular (width 0.25 m) or H-shaped (as girder DLT 1.3 in Figure 9). The right and left spans had a different load capacity by design (different stirrup diameter). The load tests were performed either by to point load slightly off-center on each span or by loads distributed along a line parallel to the girder. The experiments were monitored by load cells, strain gages, LVDTs, digital image correlation, and other techniques. Details are described in [20]. 

### 3.2. Ultrasonic Set-Up

**Figure 10:**
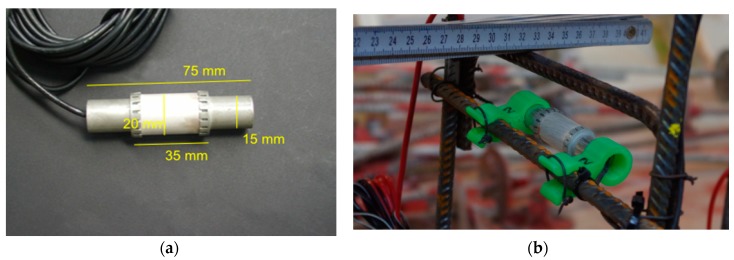
(**a**) Custom-designed ultrasonic transducer ACS S0807 for embedment into concrete; (**b**) Transducer mounted to a reinforcement cage using 3D-printed adapters.

**Figure 11:**
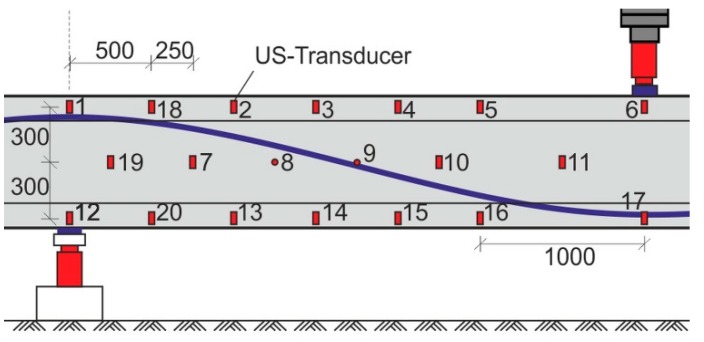
Position of embedded ultrasonic transducers in the right span of girder DLT 1.3 (dimensions in mm).

To evaluate the capability of ultrasonic monitoring to map the stress field and to identify cracked areas, the right (stronger) span of several girders was equipped with a network of embedded ultrasonic transducers ACS S0807 (Figure 10 and Figure 11). This type of transducer has been characterized and successfully applied in several experiments [21]. Girder DLT 1.3, which was used in our main experiment, contained dense network of 20 transducers that worked as transmitters and receivers. The other girders featured a less-dense network (10 transducers distributed over the same area at most). The transducers cover less than 1% of the area in any cross-section. Rebar or tendon ducts are not affected. We do not expect a significant influence on load capacity in the experiments described here. The main measurement parameters are listed in Table 1.

Data was acquired between all 48 pairs of nearest neighbors (vertical, horizontal and diagonal). All time series acquired have been processed using the scheme discussed in Section 2.2 and evaluated for velocity change and correlation using the stepwise CWI stretching technique (Section 2.3.2 and Section 2.4). 

## 4. Results

For each load step, the cumulative velocity changes compared to the beginning of the experiment (zero load) was determined for all 48 transducer pairs, allocated to the mid-point of the respective pair and interpolated over the area covered, resulting in a 2D distribution of velocity change. For the sake of consistency with previous reports and publications, we have used the negative velocity change as a parameter for the following figures.

**Figure 12:**
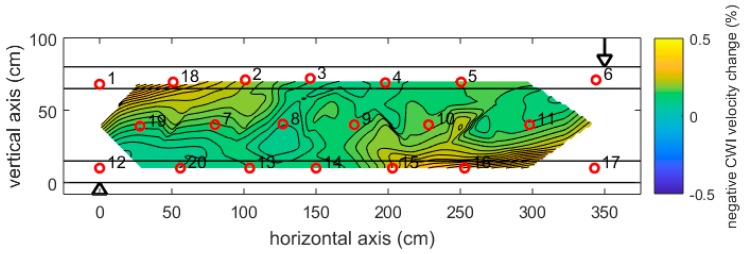
Girder DLT 1.3: Velocity change at 250 kN load, revealed by step-wise CWI and interpolations between positions of transmitter receiver pairs. Arrow: Loading point. Triangle: central support.

Figure 12 shows the distribution of velocity change at a relatively small load of 250 kN (about 13% of the failure load). The highest changes (about −0.5%) are visible in the top left and bottom right, opposite to the loading point (arrow) and central support (triangle). These are the expected zones of tension in the girder. Note, that no cracks have been detected at this stage.

**Figure 13:**
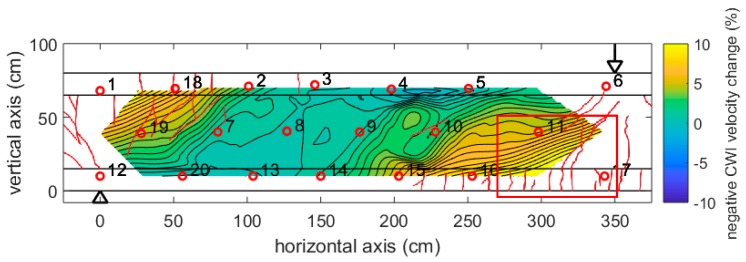
Girder DLT 1.3: As Figure 11, but 1250 kN load. Red lines: cracks visible at surface. Red rectangle: surface covered by paint for digital image correlation (no cracks visible). Note: different color scale for velocity change.

**Figure 14:**
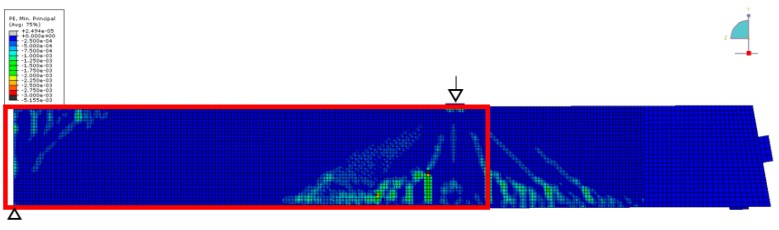
Girder DLT 1.3: Simulation of main compressive strain at 1250 kN load. Abaqus, M. Herbrand, RWTH Aachen University. Red rectangle: area displayed in Figure 12.

**Figure 15:**
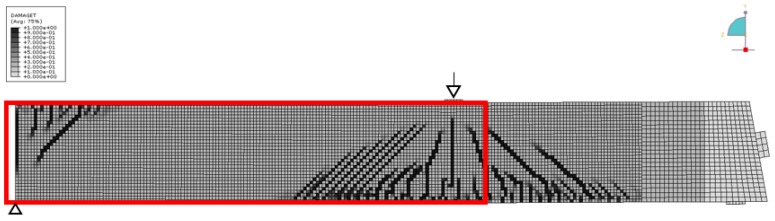
Girder DLT 1.3: Simulation of cracks (“damage parameter”) at 1250 kN load. Abaqus, M. Herbrand, RWTH Aachen University. Red rectangle: area displayed in Figure 12.

Figure 13 shows the results at 1250 kN load. Note that the color scale had to be extended by a factor of 20. Negative velocity changes up to −8% have been recorded. The figure contains information about visible crack patterns at this stage (red lines), except a rectangular area where the surface was covered by a layer of paint for digital image correlation (not discussed here). The shape of the crack patterns correlates with the shape of the negative velocity changes (indication of tension). For comparison, Figure 14 and Figure 15 show the result of Finite Element (FE) simulation (ABAQUS). Note, that only one-half of the girder has been modeled for symmetry reasons. In Figure 14, the main compressive strain is displayed, showing triangular zones with localized negative strain originating at the loading point and the central support. Figure 15 (damage parameter, i.e., cracks) shows a similar distribution. The size and shape of the tensioned/damages zones closely matches the velocity changes in the experiment (Figure 13), except a limited size anomaly between transducers 9 and 10. As a crack appears close to that location, this might reflect a feature in the girder or the experiment deviating from the idealized FE-model. This shows the sensitivity of the ultrasonic monitoring to subtle features in the stress distribution.

**Figure 16:**
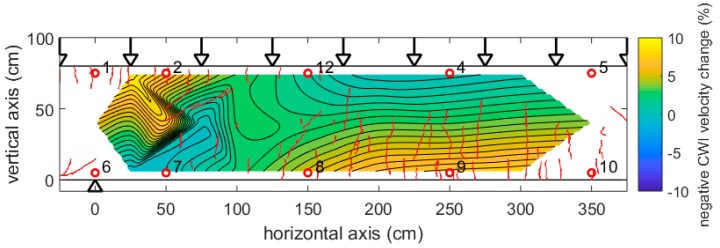
Girder DLT 1.5: Velocity change due to line load (170 kN at each point), revealed by step-wise CWI and interpolations between positions of transmitter receiver pairs. Arrow: Loading points. Triangle: central support. Red lines: cracks visible at surface.

Figure 16 shows the results of a similar girder subjected to a different load regime. The point load was replaced by a large set of loads distributed along a line on the girder, reflecting the traffic loads on a bridge in a way that is more realistic. In this girder, we had used a much-sparser network of transducers.

As expected, the pattern of velocity changes is different to Figure 12 and Figure 13, reflecting on the different load regime and stress distribution. The right part of the girder shows an approximately horizontal distribution of velocity change. The highly negative values at the bottom are due to the tension in this part of the girder, resulting in vertical cracks. The left part is influenced by the central support, leading to tension (and again highly negative velocity change) at the center and top, as well as diagonal cracks in the same area. The anomalous velocity changes below transducers 1 and 2 (nose-like shape from top right to bottom left) are potentially due to hidden cracks, influencing the ultrasound propagation left-right and top-bottom in a different way (macroscopic anisotropy). Note, that the cracks might also change the coupling conditions of the transducers, resulting in additional changes in the waveforms.

## 5. Discussion

Our research shows that CWI is a very sensitive tool that detects subtle changes in concrete by ultrasonic monitoring. The range of application can be extended to cases with higher changes by using a step-wise procedure, which has also been successfully used in [22]. The distribution of changes imaged by a very simple approach reflected the pattern expected by engineering experience and FE modeling. The largest changes have been in the zones of tension (high negative velocity change). A reduction of velocity in zones of tension is in accordance with previous research [5,7,10,13]. More sophisticated imaging approaches (as in [6]) may reveal more details and may lead to possibilities of quantitative evaluation [7,23], but are currently limited to cuboid structures and are computationally expensive. 

## 6. Conclusions and Outlook

Ultrasonic monitoring with embedded transducers, if evaluated appropriately, can contribute to the identification of stress patterns and cracking before visual signs appear on the surface in large-scale load experiments and potentially also at real structures. This also works with sparse transducer networks (about 1 m distance between transducers) and very simple imaging procedures.

For field applications, it would be desirable to develop imaging procedures, which are more quantitative than our approach, but less computationally expensive than the others. However, the main issue as in many monitoring technologies would be the separation of influence factors affecting ultrasonic wave propagations, as temperature, moisture and various damage mechanisms. When these issues are solved, implementation on selected real structures might help to make decisions in infrastructure asset management. Ultrasonic monitoring might help to identify zones with an elevated stress level or progressing damage, covering a significant volume with a limited number of sensors, The results might—in combination with other measures—trigger more detailed non-destructive and/or destructive investigations as well as decisions on rehabilitation, strengthening or load restrictions. 

## Figures and Tables

**Table 1:** Ultrasonic measurement parameters.

Parameter	Value
Number of transducers	10–20
Number of transmitter-receiver-combinations	24–48
Distance transmitter-receiver	0.3–2.0 m
Central frequency	60 kHz
Signal shape	rectangle
Sample frequency	1 MHz
Recording time	5 ms
Averaging	1–12
Measurement interval	2 min
