# Correction: Niederleithinger, E., et al. Processing Ultrasonic Data by Coda Wave Interferometry to Monitor Load Tests of Concrete Beams. *Sensors* 2018, *18*, 1971

**DOI:** 10.3390/s19010147

**Published:** 2019-01-03

**Authors:** Ernst Niederleithinger, Xin Wang, Martin Herbrand, Matthias Müller

**Affiliations:** 1 Bundesanstalt für Materialforschung und -prüfung (BAM), Unter den Eichen 87, 12205 Berlin, Germany; xin.wang@bam.de; 2 RWTH Aachen University (now WTM Engineers GmbH), Templergraben 55, 52062 Aachen, Germany; martin.herbrand@outlook.de; 3 Bundesanstalt für Straßenwesen (BASt), Bruederstr. 53, 51427 Bergisch Gladbach, Germany; muellerm@bast.de

The authors wish to make the following corrections to this paper [1]:

## 1. Change in Figure

The authors wish to make the following correction to Figure 6. Because of the misplacement of a graphical element, replace:


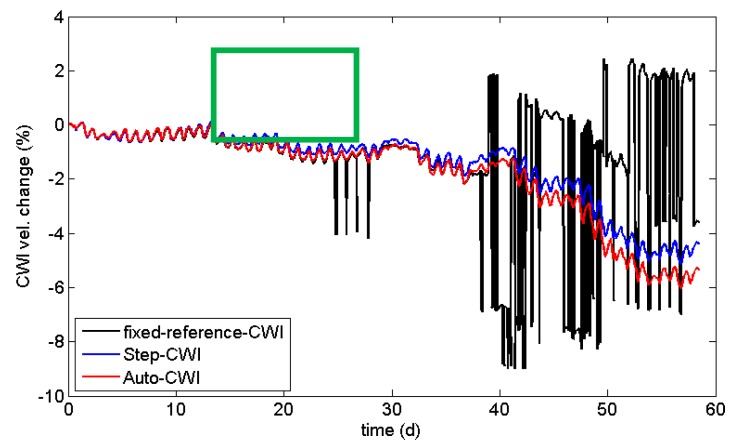


with


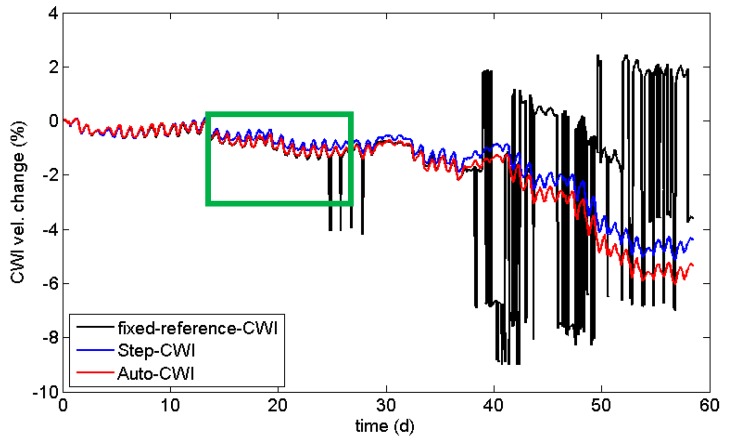


## 2. Change in Funding

The authors wish to insert this additional sentence in the Funding section:

“Xin Wang is funded by the H2020-MSCA-ITN-2015 Framework Programme Project N°676139 “Infrastar” of the European Union.”

The authors would like to apologize for any inconvenience caused to the readers by these changes.
